# Comprehensive analysis of faecal metagenomic and serum metabolism revealed the role of gut microbes and related metabolites in detecting colorectal lateral spreading tumours

**DOI:** 10.1080/21505594.2025.2489154

**Published:** 2025-04-13

**Authors:** Hao Lin, Yudai Chen, Ming Zhou, Hongli Wang, Lichun Chen, Li Zheng, Zhixin Wang, Xiaoling Zheng, Shiyun Lu

**Affiliations:** a Shengli Clinical Medical College, Fujian Medical University, Fuzhou, China; b Department of Gastroenterology, Fujian Provincial Hospital, Fuzhou, China; c Department of Gastroenterology, Fuzhou University Affiliated Provincial Hospital, Fuzhou, China; d Department of Digestive Endoscopy, Fujian Provincial Hospital, Fuzhou, China

**Keywords:** Colorectal lateral spreading tumours, gut microbiota, metabolomic, choline metabolism in cancer, amino acid metabolism

## Abstract

Colorectal lateral spreading tumours (LST), early-stage lesions of colorectal cancer (CRC), are associated with gut microbiota dysbiosis. However, the functional alterations in gut microbiota and their metabolic pathways remain inadequately understood. This study employed propensity score matching to compare 35 LST patients with 35 healthy controls. Metagenomic and metabolomic analyses revealed notable differences in gut microbiota composition and metabolic pathways. LST patients exhibited a marked reduction in short-chain fatty acid (SCFA)-producing probiotics, such as *Roseburia*, *Clostridium*, and *Butyricicoccus sp-OF13–6*, alongside anti-inflammatory metabolites. In contrast, potential intestinal pathogens linked to inflammatory bowel disease (IBD), including *Escherichia* and *Citrobacter amalonaticus*, were significantly enriched. Orthogonal partial least squares discriminant analysis (OPLS-DA) highlighted significant metabolic disparities between the groups, with enrichment in pathways associated with cholesterol metabolism, choline metabolism in cancer, and amino acid metabolism – all relevant to cancer progression. Key biomarkers identified for LST included fumarate, succinate, glutamic acid, glycine, and L-aspartic acid, which were closely linked to these pathways. Functional studies demonstrated that these metabolites promoted the proliferation and invasion of HCT-116 and SW480 human colorectal cancer cells *in vitro*. Metagenomic and metabolomic analysis revealed a strong positive correlation between *Escherichia* and *Ruminococcus sp-AM41-2AC* abundance and the enriched pathways, whereas reductions in *Roseburia species*, including *Roseburia-OF03–24* and *Roseburia intestinalis_CAG13*—exhibited negative correlations. These results suggest that gut microbiota and metabolite alterations in LST contribute to intestinal inflammation and CRC development, underscoring their potential as biomarkers for early detection and therapeutic targets.

## Introduction

Colorectal cancer (CRC) is the third most frequent malignancy and a major cause of death worldwide [1]. In China, over 500,000 people are diagnosed with CRC each year with approximately 300,000 CRC-related deaths [2]. CRC has been increasingly common among young people in recent years. Clinical research for the prevention and treatment of CRC is urgently needed. Early diagnosis and therapy are critical to improving the prognosis of CRC patients and lowering the disease burden in the population [3,4]. Lateral spreading tumours (LST) are raised lesions that originate from the colon mucosa and have a diameter larger than 10 mm [5]. They mainly spread superficially along the mucosal surface and seldom invade the deeper region of the intestinal lumen. LST is an important pre-cancerous lesion in CRC [6]. Studies have shown a close relationship between LST and CRC. Dynamic tracking has revealed that LST can develop into progressive CRC within three years [7,8]. When the diameter of LST exceeds 20 mm, the chance of cancer can reach 46% [9]. LST accounts for about 17.2% of all high-risk adenomas of the colorectum, and the incidence of mucosal invasive carcinoma ranges from 2.6% to 12.3% [10,11]. Therefore, exploring the symptoms of LST lesions might offer a critical foundation for CRC prevention.

Gut microbiota can provide energy for the development and maturation of intestinal cells, help in synthesizing vitamins and substances metabolism, stimulate intestinal lymphocyte immune function, inhibit the colonization of pathogenic bacteria, and maintain numerous basic physiological functions in the human body [12]. In recent years, there has been a lot of interest in the relationship between gut microbiota and CRC pathogenesis. Numerous studies have found that the gut microbiota of CRC patients differs considerably from that of the healthy population [13]. Changes in the proportion and stability of certain probiotics, such as (*Lactobacillus acidophilus* and *Bifidobacterium longum*) affect the microecological balance of the host’s colonic epithelium, thereby preventing or contributing to the formation and development of CRC [14,15]. Studies have revealed that modulating gut microbiota might help prevent and treat colon cancer to some extent. The use of probiotics can alter malignant biological behaviours, such as proliferation, apoptosis, and adhesion of colon cancer cells [16,17]. Furthermore, it has also been shown that gut microbiota can cause an inflammatory response in the intestinal mucosa, leading to increased intestinal mucosal epithelial damage [18]. Investigating the alterations in gut microbiota in LST patients might indirectly prevent CRC.

Tumours cause a change in metabolic state, and metabolic analysis can efficiently detect tumours. The CRC-related abnormal metabolites have been reported to be biomarkers for the diagnosis of CRC [19]. Certain gut microbial metabolites, such as β-glucosidase and azoreductase, can cause cancer, stimulate the synthesis of dimethylhydrazine and nitrite, and induce intestinal carcinogenesis [20]. In the patients with bowel cancer, the serum levels of L-valine and L-threonine were modest; however, the 3-hydroxybutyric acid level was dramatically elevated. These biomarkers serve as a foundation for CRC detection [21]. Numerous metabolic processes, including glycolysis, the TCA cycle, the urea cycle, pyrimidine, tryptophan, and polyamine metabolism, and gut microbiota-host co-metabolism, are dysregulated in CRC patients. A diagnostic model with seven metabolites was constructed in previously published studies [22,23].

Considering a direct relationship between LST and CRC, medical researchers have conducted extensive research on LST in recent years, with most of the studies focusing on pathological symptoms [11], diagnostic modalities [24], and therapeutic modalities, such as endoscopic treatment or surgery [25,26]. The changes in gut microbiota as well as serum metabolism in LST patients have not been investigated yet. This study aimed to thoroughly investigate the changes in gut microbiota and serum metabolism in LST patients in order to identify biomarkers that could distinguish LST from the healthy population as well as lay a theoretical foundation for using gut microbiota-serum metabolites (GM-SMs) in the diagnosis of LST and prevention of CRC.

## Materials and methods

### Sample collection

The study protocol was approved by the Fujian Province Hospital (Fujian, China) Committee (K2022-09-027/02). The 85 healthy individuals and 63 LST patients (aged 25 to 75 years) were obtained from Fujian Province Hospital. The basic information of the participants is summarized in Table 1. The selection criteria were as follows: (1) colon confirmed as LST based on the colonoscopy results validated by two experts in the digestive endoscopy facility; (2) no probiotics, prebiotics, or antibiotics had been used in the previous month; (3) the patients with diseases affecting gut microbiota, such as cirrhosis, chronic pancreatitis, diabetes, hyperlipidaemia, etc. were excluded; (4) no history of abdominal surgery or participation in other clinical trials; (5) pregnant or lactating women were excluded; and (6) the patients with end-stage diseases were excluded. All the participants completed informed consent forms. To reduce the effect of confounding bias in the baseline data, propensity score matching (PSM) was used to match the above LST and HC groups based on gender, age, and BMI to balance the differences between the groups. The final collection of faeces and serum were collected from 35 health and 35 LST patients. Before treatment, the faecal samples of the patients were collected in a sterile EP tube and stored at −80°C. A total of 2 mL venous blood was collected from the patients and healthy individuals into an ethylenediaminetetraacetic acid (EDTA) anticoagulant tube. The blood was centrifuged at 4°C and 3000 × g for 10 min, and the separated serum was stored at −80°C.

**Table 1.** Clinical characteristics and basic information of study populations.

Characteristics	HC (*n* = 35)	LST (*n* = 35)	*P*-value
Male, n (%)	9(25.7%)	12 (34.3%)	0.434
Age (mean, SD)	54.5(11.9)	52.3(12.3)	0.892
Laboratory index, median			
ALT (U/L)	16.1(9.0–25.0)	20.9(9.0–91.0)	0.077
AST (U/L)	19.1(10.0–28.0)	19.3(11.0–45.0)	0.888
Triglycerides (mg/dL)	1.2(0.2–3.9)	1.8(0.6–6.9)	0.028
Total cholesterol (mg/dL)	4.8(3.2–7.3)	5.1(2.9–7.4)	0.337
Albumin (g/L)	43.7(39.0–51.0)	44.8(35.0–52.0)	0.183
Glycated hemoglobin (mmol/L)	5.6(4.3–8.0)	5.6(4.6–7.8)	0.857

### Fecal DNA extraction and metagenomic sequencing

The Fast DNA^TM^ Fecal Genomic DNA Extraction Kit (QIAGEN, Germany) was used to extract the total DNA of gut microbiota. DNA purity was tested using Nanodrop, and DNA integrity was analysed using 1% agarose gel electrophoresis. The DNA samples that passed the test were arbitrarily broken down into around 300-bp fragments using a Covaris ultrasonic crusher. The fragmented DNA samples were then sent to Beijing Ovison Gene Technology Co., Ltd. (Beijing, China) to generate a metagenomic sequencing library. Once the library was generated and passed quality control, it was tested using an Agilent 2100 and sequenced on an Illumina NovaSeq PE150 platform. Then, the contaminated sequence reads with a high degree of similarity were removed by aligning the raw sequence reads with the host genome using BWA (Version 0.7.9a).

### Metagenomic data analysis

The optimized sequences were spliced and assembled using Megahit (Version 1.1.2) software, and open reading frame (ORF) prediction was performed using Prodigal. The predicted gene sequences were clustered using CD-HIT (Version 4.6.1) software to construct a non-redundant gene set. Using Diamond (Version 0.8.35), the amino acid sequences of the non-redundant gene set were compared with the Kyoto Encyclopedia of Genes and Genomes (KEGG) database (Version 94.2) to obtain the corresponding KEGG ontologies of the genes. The abundance of an ontology was calculated using the sum of the abundance of the genes corresponding to the ontology. Borrowed module analysis to identify key functional genes. Alpha (α)-diversity was assessed using Shannon and Simpson indices. Beta diversity was calculated using principal coordinate analysis (PCoA) based on the Bray-Curtis distance matrix. The statistical differences in α-diversity and microbial taxa among all the groups were assessed using the Mean-Whitney-Wilcoxon test. The original data were uploaded at NCBI (https://www.ncbi.nlm.nih.gov/bioproject/PRJNA1212478).

### Fecal and serum metabolomic assays

The faecal samples were thawed on ice. A 100 mg sample was added to 1 mL of pre-cooled methanol-acetonitrile-water solution (2:2:1, v/v/v) extract. The sample was homogenized using an MP homogenizer operating at 60 hz for 60 sec, followed by two 30-min sonication at 4°C. After holding the temperature at −20°C for 60 min, the supernatant was extracted by centrifugation at 13,000 × g and 4°C for 15 min. The supernatant was then vacuum-dried and added to 100 μL of a 50% (v/v) aqueous acetonitrile solution. This mixture was vortexed and centrifuged at 14,000 × g and 4°C for 15 min, and the supernatant was then poured into injection vials using a 0.22-μm membrane filter to prepare for the next assay.

The serum samples were thawed at 4°C. A 100 µL sample was mixed with 400 µL of pre-cooled methanol-acetonitrile solution (1:1, v/v) extract, vortexed, and incubated at −20°C for 1 h to precipitate the proteins. After centrifugation for 20 min at 14,000 × g and 4°C, the supernatant was extracted and vacuum-dried. The dried residue was dissolved in 50% (v/v) acetonitrile in water and then centrifuged at 14,000 × g for 15 min at 4°C. The quality control (QC) samples were prepared by combining 10 µL of supernatant from all the samples for subsequent testing. Ultra-High Performance Liquid Chromatography- Mass Spectrometry (UHPLC-MS) was used for the detection of metabolites in faecal and serum samples. ACQUITY UPLC BEH Amide liquid chromatography column (Water, UK) on a UPLC system (SCIEX, UK) were used to analyse the composition of the analyte. Furthermore, samples were detected using the TripleTOF5600 + mass spectrometer (SCIEX, USA) in positive and negative ion modes. The mobile phase B was made up of 100% Acetonitrile, while mobile phase A was made up of 25 mm ammonium hydroxide and 25 mm ammonium acetate in water. The mobile phase conditions for liquid chromatography were as follows: 95% mobile phase B for 0.5 min; mobile phase B decreased from 95%–65% during 0.5–7 min; mobile phase B decreased from 65%–40% during 7–8 min; mobile phase B maintained at 40% during 8–9 min; mobile phase B increased from 40%–95% during 9–9.1 min; and mobile phase B maintained at 95% from 9.1–12 min.

### Metabolomic data analysis

The peaks were detected, extracted, aligned, and integrated using an XCMS-based self-writing R package and annotated using the MS2 database from Allwegene (Beijing, China). All the analyses were performed using the R package from Metabo Analyst R (https://github.com/xia-lab/MetaboAnalystR). Stacked graphs and heatmaps were drawn using MicrobiomeAnalyst (https://www.microbiomeanalyst.ca/) and ImageGP (https://www.bic.ac.cn/BIC/#/), respectively. The R package ropls (v1.6.2) was used for orthogonal partial least squares-discriminant analysis (OPLS-DA) determine the overall metabolite differences between groups and the degree of variability between samples within groups. The identified metabolites were annotated using the KEGG Compound Database (http://www.kegg.jp/kegg/compound/), followed by mapping to the KEGG Pathway Database (http://www.kegg.jp/kegg/pathway.html). The differential metabolites were identified based on projected variable importance (VIP) ≥1.0, absolute fold change (FC) ≥2.0, and *p* < 0.05. The Mann-Whitney-Wilcoxon test was used for between-group comparisons.

### Validation in vitro cell culture

Three significantly increased and three significantly decreased metabolites from the LST group were selected to validate their effects on cellular activity and invasive potential in vitro. Human colorectal cancer cell lines HCT-116 and SW480 were obtained from the Cell Bank of the Chinese Academy of Sciences (Shanghai, China). Both cell lines were cultured in Dulbecco’s Modified Eagle Medium (DMEM) supplemented with 10% foetal bovine serum (FBS) and 1% penicillin-streptomycin, and maintained at 37°C in a humidified atmosphere with 5% CO_2_.

The impact of differential metabolites on cancer cell proliferation was evaluated using the 3-(4,5-dimethyl-2-thiazolyl)-2,5-diphenyl-2-H-tetrazolium bromide (MTT) assay. Cells (100 μL) were seeded into 96-well plates at a density of 3.5 × 103 cells/well. The blank group contained DMEM medium alone. After 24 hours of incubation at 37°C with 5% CO2, the medium was replaced with 100 μL of metabolites at a final concentration of 500 μM in the treatment group. The blank and control groups received 100 μL of DMEM. Following 48 hours of incubation, the medium was aspirated, and 100 μL of fresh DMEM and 100 μL of 0.5% MTT solution were added to each well. The plates were incubated at 37°C for 4 hours. The supernatant was then removed, and the precipitates were dissolved in 200 μL of DMSO. Absorbance was measured at 570 nm using a microplate reader, and cell viability was calculated as:

Cell viability (%) = [(OD treated – OD blank)/(OD control – OD blank)] × 100%.

The effects of differential metabolites on cell migration and invasion were assessed using the Transwell chamber assay. Matrigel was diluted with serum-free, pre-cooled Roswell Park Memorial Institute (RPMI) 1640 medium at a 1:8 ratio to prepare a 10 mg/mL gel. A total of 100 μL of the diluted Matrigel was evenly spread on the bottom of the Transwell chamber and allowed to gel at 37°C for 4 hours. After gelation, 200 μL of HCT-116 and SW480 cell suspensions, treated with serum-free medium, were added to the upper chamber. Simultaneously, 500 μL of RPMI 1640 medium containing 10% FBS was added to the lower chamber. Each group was tested in triplicate and incubated for 24 hours. The chambers were then washed with PBS, and non-migratory cells adhering to the upper surface of the Transwell membrane were gently removed using sterile cotton swabs. The lower chambers were fixed with paraformaldehyde, stained with haematoxylin and eosin (HE), and sealed. Cellular invasion was quantified by counting cells in five randomly selected fields at 100× magnification under an inverted microscope.

### Statistical analysis

#### Propensity score matching (PSM) analysis

In this study, 63 LST patients and 85 healthy volunteers were collected from May 2023 to August 2023. In order to reduce the effect of confounding bias of baseline information, propensity score matching function (PSM) was used for matching by SPSS 26.0. The control and LST groups were 0/1 categorical variable treatment indicators, age, gender, and BMI were used as covariates with a 1:1 matching degree and a caliper value of 0.02, and the PS value was calculated, and finally the cases with the closest scores were matched. Thirty-five LST patients were matched 1:1 with controls, and the remaining 28 LST patients were not successfully matched and the data were excluded.

#### Clinical variables

Statistical analyses were conducted using SPSS 26.0 software. Normally distributed quantitative data were expressed as mean ± SD, while the non-normally distributed quantitative data were defined by medians and quartiles. Two-sample t-tests were employed to compare two groups. Differences with *P*-values <0.05 were considered statistically significant. Spearman’s correlation coefficient was used to conduct a univariate correlation analysis of the differential gut metabolites and microbiota. The correlation heatmaps and network between differential metabolites and differential microbiota as well as those of differential metabolites with functionally enriched pathways were identified using a *P*-value of < 0.05.

## Results

### Basic information and clinical characteristics of study populations

**Figure 1.**
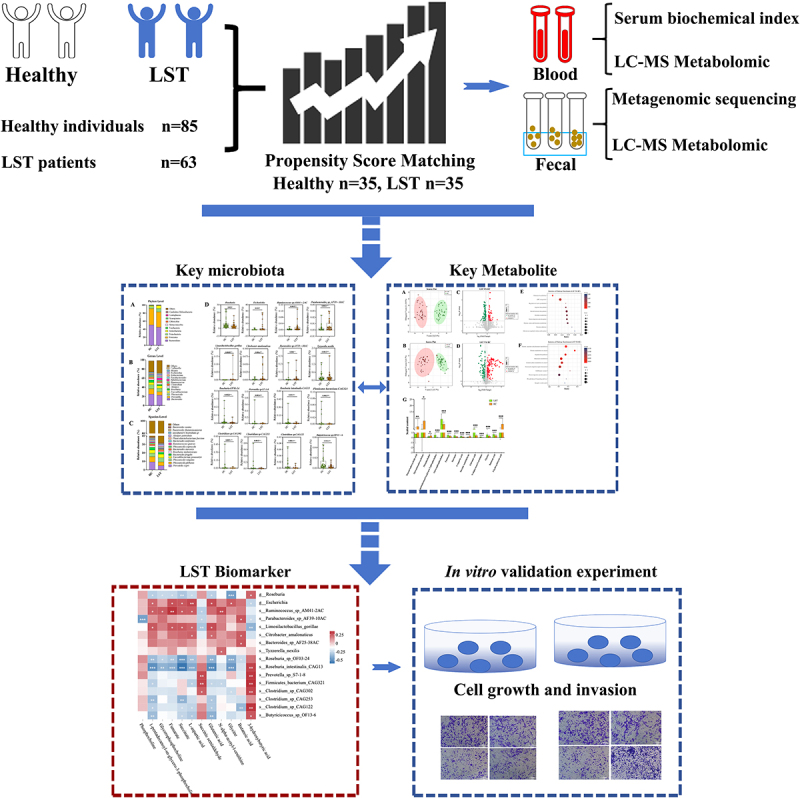
Research flowchart diagram of this study.

To explore the pathogenesis of LST, 63 patients with LST and 85 healthy individuals were enrolled (Figure 1), and collected basic information on gender, BMI, hypertension, diabetes mellitus, family history of colorectal cancer, smoking history, and alcohol consumption (Supplementary material 1). To reduce the effect of confounding bias of the baseline information, 1:1 PSM matching was performed with the LST group as the baseline, and a total of 35 pairs of LST and HC groups were successfully matched. Clinical serum biochemical indexes were analysed by t-test to obtain a positive correlation between LST and TG levels. Age, gender, alanine aminotransferase (ALT), aspartate aminotransferase (AST), total cholesterol (TC), albumin, and glycated haemoglobin levels did not show differences between the groups (Table 1).

### Diversity and composition of gut microbiota in the patients with LST

**Figure 2.**
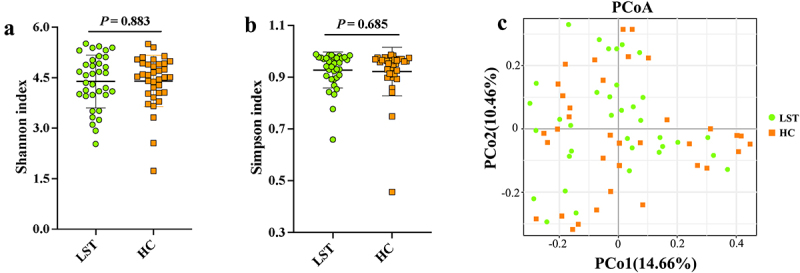
Gut microbial diversity in LST patients. (a) Shannon index (b) Simpson index; (c) Principal coordinates analysis (PCoA) based on Bray-Curtis distance between the LST and HC groups.

Metagenomic sequencing analyses were performed to explore the relationship between gut microbiota and LST. A relative decrease was detected in the alpha diversity in the LST group using the Shannon and Simpson indices; however, the difference was not significant as compared to the healthy control (HC) group (Figure 2(a,b)). In addition, PCoA based on the Bray-Curtis distance revealed that the structure of gut microbiota in the LST patients differed slightly from that of healthy individuals (Figure 2(c)).

**Figure 3.**
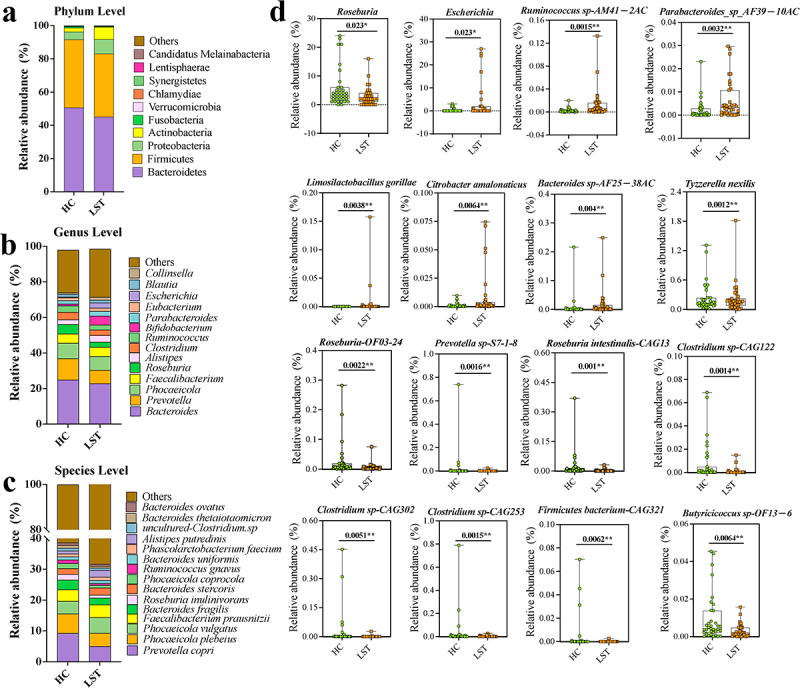
Gut microbial composition at the (a) phylum level, (b) genus level, and (c) species level. (d) Relative abundances of the identified bacteria with significant changes between LST and HC groups. *p < 0.05 and **p < 0.01.

Considerable differences in gut microbial profiles were observed between the LST and HC groups from the phylum to the species level. At the phylum level, Bacteroidetes, Firmicutes, and Proteobacteria were the dominant phyla; the LST group showed an increase in the abundance of Proteobacteria and a decrease in that of Bacteroidetes as compared to the HC group; however, the difference was not significant (Figure 3(a)). The top 20 abundant bacterial genera in each group are shown in Figure 3(b). As compared to the HC group, the abundance of *Roseburia* significantly decreased, while that of *Escherichia* significantly increased in the LST group (*p* < 0.05). At the species level, a total of 10,110 identified species were detected in the two groups (Figure 3(c)). Among the determined species with abundances greater than 0.01%, *Ruminococcus sp-AM41 − 2AC*, *Parabacteroides sp-AF39 − 10AC*, *Limosilactobacillus gorillae*, *Citrobacter amalonaticus*, and *Bacteroides sp-AF25-38AC* significantly increased, while *Tyzzerella nexilis*, *Roseburia-OF03–24*, *Roseburia intestinalis-CAG13*, *Prevotella sp-S7-1-8*, *Firmicutes bacterium-CAG321*, *Clostridium sp-CAG302*, *Clostridium sp-CAG253*, *Clostridium sp-CAG122*, and *Butyricicoccus sp- OF13–6* showed a significant decrease in the LST group as compared to the HC group (Figure 3(d)).

### Lst-related microbial function prediction

**Figure 4.**
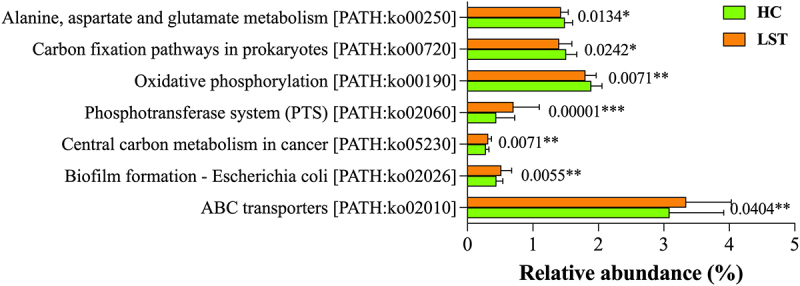
Functional annotation of differential microbiota of the LST and HC groups based on KEGG pathways. *p < 0.05, **p < 0.01, and ***p < 0.001.

In order to study the role of differential microbiota in LST, functional annotation was conducted using the KEGG database. The primary metabolic pathways of the LST group included ABC transporter metabolic pathway (ko02010), biofilm formation-*Escherichia coli* (ko02026), central carbon metabolism in cancer (ko05230), and phosphotransferase system (ko02060). In contrast, the LST group had a considerably lower enrichment of the oxidative phosphorylation (ko00190), carbon fixation pathway in prokaryotes (ko00720), and alanine, aspartate, and glutamate metabolism (ko00250) pathways (Figure 4).

**Figure 5.**
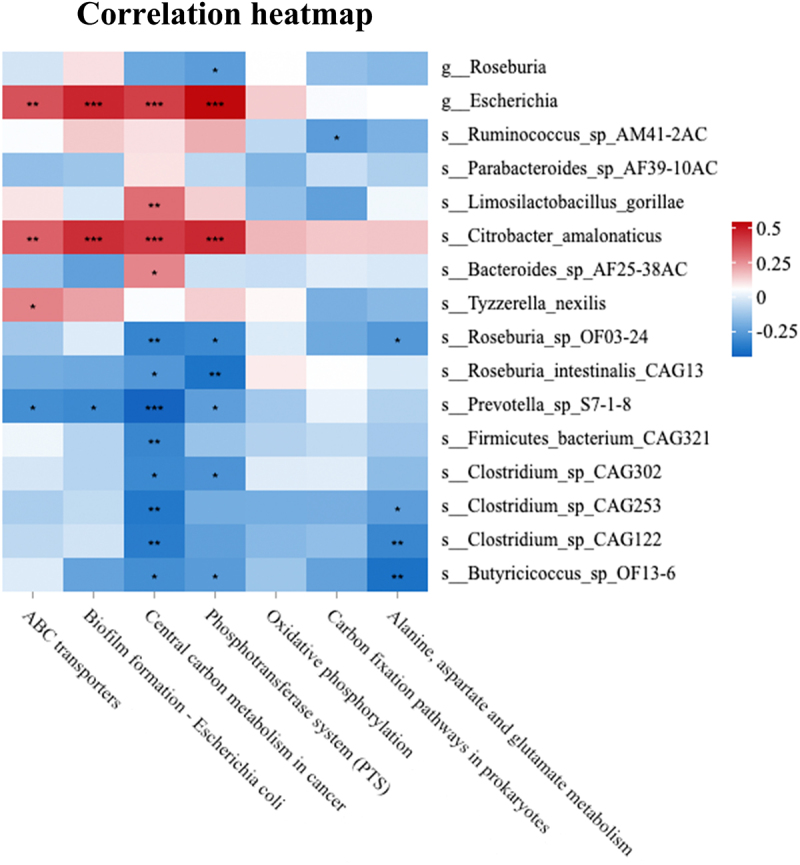
Spearman’s correlation analysis of significantly altered gut microbiota and KEGG pathways. *p < 0.05, **p < 0.01, and ***p < 0.001.

Spearman’s correlation analysis showed that the abundance of *Escherichia* and *Citrobacter amalonaticus* in the LST group was positively correlated with the ABC transporter metabolic pathway, biofilm formation-*Escherichia coli*, central carbon metabolism in cancer, and phosphotransferase system (PTS) pathways (*p* < 0.01 and *p* < 0.001, respectively). *Roseburia*, *Roseburia-OF03–24*, *Roseburia intestinalis-CAG13*, *Prevotella sp-S7-1-8*, *Firmicutes bacterium-CAG321*, *Clostridium sp-CAG302*, *Clostridium sp-CAG253*, *Clostridium sp-CAG122*, and *Butyricicoccus sp- OF13–6* exhibited lower abundances and were significantly negatively correlated with the above pathways. In particular, central carbon metabolism in cancer was extremely significantly correlated with these bacterial strains (*p* < 0.001). *Roseburia-OF03–24*, *Clostridium sp-CAG253*, *Clostridium sp-CAG122*, and *Butyricicoccus sp- OF13–6* showed significantly negative correlations with the alanine, aspartate, and glutamate metabolism (*p* < 0.05 and *p* < 0.01) (Figure 5).

### Fecal and serum metabolites in the LST patients

**Figure 6.**
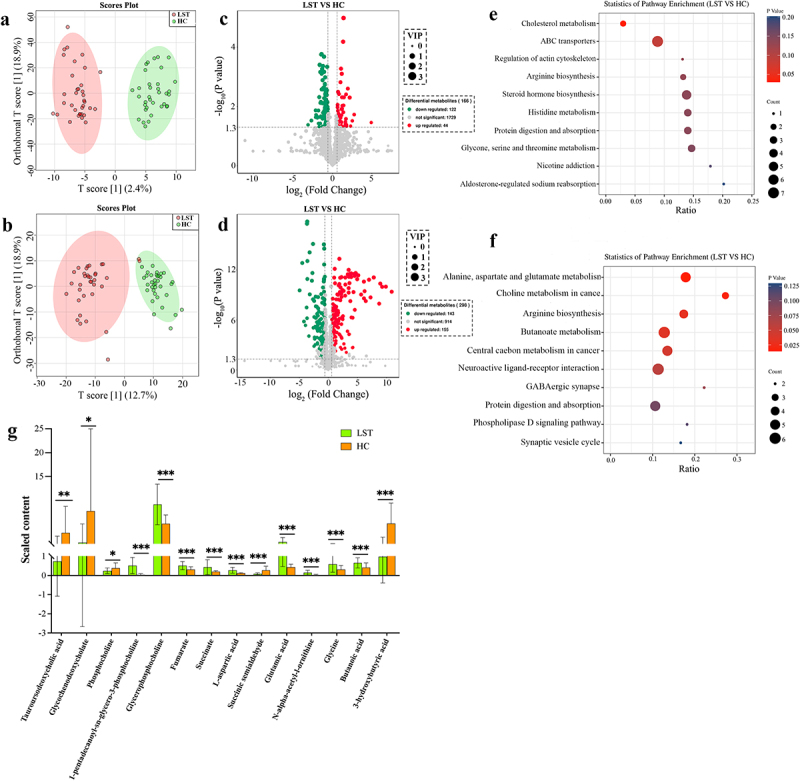
Fecal and serum metabolites in the LST group. Orthogonal partial least squares discriminant analysis (OPLS-DA) of the (a) fecal and (b) serum metabolites. Volcano plot of differential metabolite in (c) fecal and (d) serum samples. Enrichment analysis of (e) fecal and (f) serum metabolites in KEGG pathways. (g) Comparison of significantly different metabolite enrichment in pathways between the LST and HC groups. *p < 0.05, **p < 0.01, and ***p < 0.001.

In order to further investigate the interaction between the LST-related microbiota and the host, the faecal and serum metabolites were analysed using UHPLC-MS/MS. Both the score plots of OPLS-DA revealed a stronger separation between the HC and LST groups as well as improved sample repeatability within the two groups, suggesting significantly different metabolites between the two groups (Figure 6(a,b)). In total, 1,895 metabolites were identified in the faecal samples, among which, 166 were differentially expressed in the LST group, including 44 up-regulated and 122 down-regulated (Figure 6(c)). The faecal differential metabolites were highly enriched in cholesterol metabolism; A total of 10 metabolites are detected in this pathway, of which taurochenodeoxycholate and glycochenodeoxycholate were the most important differential metabolites in this pathway (Figure 6(e)).

A total of 1,212 metabolites were identified in the serum samples, including 298 distinct, 155 up-regulated, and 143 down-regulated metabolites in the LST (Figure 6(d)). These differential metabolites were considerably enriched in choline metabolism in cancer (11 metabolites were enriched, 3 metabolites were significantly different), alanine, aspartate, and glutamate metabolism (28 metabolites were enriched, 5 metabolites were significantly different), arginine biosynthesis (23 metabolites were enriched, 4 metabolites were significantly different), central carbon metabolism in cancer (37 metabolites were enriched, 5 metabolites were significantly different), and butanoate metabolism pathways (47 metabolites were enriched, 6 metabolites were significantly different). Phosphocholine, 1-pentadecanoyl-sn-glycero-3-phosphochol -ine, glycerophosphocholine, fumarate, succinate, L-aspartic acid, succinic semialdehyde, glutamic acid, N-alpha-acetyl-l-ornithine, glycine, butanoic acid, and 3-hydroxybutyric acid were the important differential metabolites in these pathways (Figure 6(f,g)).

### Gut microbiota interactions with fecal and serum metabolites were related to LST

**Figure 7.**
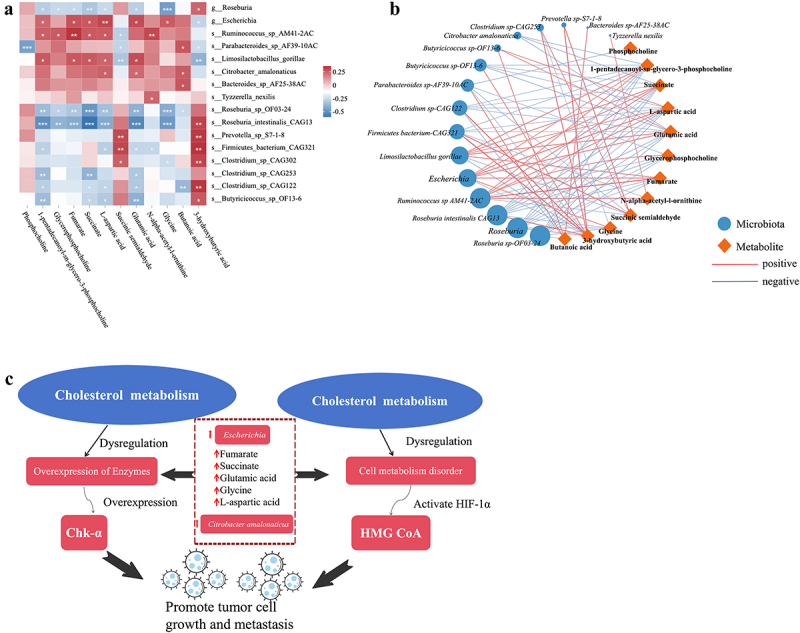
(a) Correlation heat map between the significant gut microbiota and metabolites. (b) Correlation network diagram showing significant metabolites and significant changes in bacterial taxa. (c) Intestinal microbes of LST affect key KEGG pathways and key functional genes. *p < 0.05, **p < 0.01, and ***p < 0.001. Blue and orange dots represent microbiota and metabolite, respectively. Edges between the nodes indicate Spearman’s positive (red) and negative (blue) correlations, and the edge thickness indicates the strength of the correlations. The node sizes indicate the number of related metabolites.

In order to further observe the potential roles of gut microbiota and functional metabolites in disease progression, a complete correlation heat map and network analysis were conducted using metagenomic and metabolome data (Figure 7(a,b)). Glycochenodeoxycholate was correlated negatively with *Ruminococcus sp-AM41 − 2AC* and *Parabacteroides sp-AF39 − 10AC*. The abundances of *Roseburia*, *Roseburia-OF03–24*, and *Roseburia intestinalis-CAG13* were negatively correlated with 1-pentadecanoyl-sn-glycero-3-phosphocholine, glycochenodeoxycholate, fumarate, succinate, L-aspartic acid, glutamic acid, and glycine in the LST group. On the other hand, the abundances of *Escherichia*, *Ruminococcus sp-AM41-2AC*, and *Limosilactobacillus gorillae* were significantly positively correlated with these metabolites (*p* < 0.05). *Roseburia-OF03–24*, *Roseburia intestinalis-CAG13*, *Prevotella sp-S7-1-8*, *Firmicutes bacterium-CAG321*, *Clostridium sp-CAG302*, *Clostridium sp-CAG122*, and *Butyricicoccus sp-OF13 − 6* having a marked decrease in the LST group showed that succinic semialdehyde and 3-hydroxybutyric acid (*p* < 0.05) were significantly positively correlated with these bacteria. *Escherichia*, *Ruminococcus SP-AM41-2AC*, *Roseburia intestinalis-CAG13*, *Roseburia*, and *Roseburia* OF03–24 were highly associated with different altered bacteria.

*Escherichia* and *Citrobacter amalonaticus* in the LST group significantly increased and indirectly affected the production of Fumarate, Succinate, Glutamic acid, Glycine, and L-aspartic acid metabolites, which affect intracellular oxidative stress levels, regulate Choline metabolism in cancer. To further investigate the pathogenic mechanisms of gut microbiota and metabolites in LST patients, gut metabolic modules were used to explore the key genes [27]. Enzymatically active genes in the Cholesterol metabolism pathway (e.g. *Chk-α* for Choline kinase and *HMG CoA* for cholesterol synthesis key enzyme), thereby inducing cancer cell growth (Figure 7(c)).

### Cell growth and invasion assay in vitro

**Figure 8.**
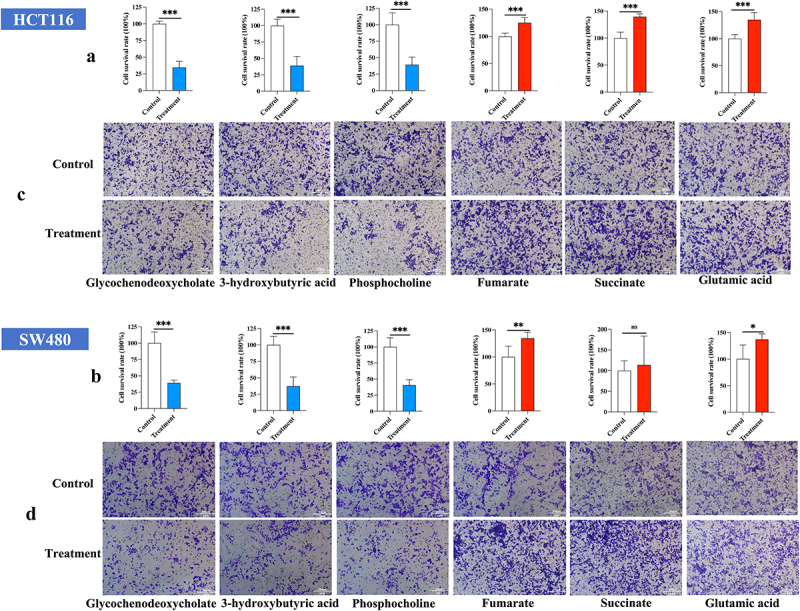
(a) Cell survival rate of significant metabolites on HCT116 cells. (b) Cell survival rate of significant metabolites on SW 480 cells. (c) The invasive and migratory abilities of the differential metabolites on HCT-116 cells. (D) The invasive and migratory abilities of the differential metabolites on SW480 cells. *p < 0.05, **p < 0.01, and ***p < 0.001.

The MTT assay revealed that the metabolites with significantly decreased in the LST group (Glycochenodeoxycholate, 3-hydroxybutyric acid, Phosphocholine) all showed highly significant inhibition of proliferation of HCT-116 cells and SW480 cells. On the contrary, metabolites increased in the LST group (Fumarate, Succinate, Glutamic acid) promoted the growth of HCT-116 cells; Fumarate and Glutamic acid and significantly promoted the growth of SW480 cells. Fumarate, in particular, reached highly significant levels of growth-promoting ability for both cancer cells (Figure 8(a,b)). The results of invasion assay also demonstrated that Glycochenodeoxycholate, 3-hydroxybutyric acid, and Phosphocholine highly significantly inhibited the invasion ability of HCT-116 cells and SW480 cells in vitro, while Fumarate, Succinate, Glutamic acid significantly induced the invasive ability of those cells (Figure 8(c,d)).

## Discussion

LST is a significant precancerous lesion of CRC [28], and the imbalance of gut microbiota plays a crucial role in the pathophysiology of CRC, which can be employed as a new method for the early detection and therapy of CRC [13]. However, the gut microbiota features in LST patients, such as disease-associated strains and functional potential and intricate interactions of gut microbiota with host metabolism, have not been fully understood yet. Therefore, the current study performed a combined analysis of the datasets, including gut microbiota and gut and serum metabolites. The LST patients have been reported to have disturbances in their gut microbiota and gut serum metabolites. The combined evaluation of gut microbiota and metabolites might be a novel tool for the LST diagnosis, thereby providing a reference value for early CRC prevention.

The disturbances in gut microbiota occur in patients with LST, and the abundance of certain essential gut-beneficial bacteria dramatically decreased in this study. The genus *Roseburia*, including species *Roseburia-OF03–24* and *Roseburia intestinalis-CAG13*, is considered a biomarker for symptomatic disorders, such as gallstones, and a probiotic to replenish the beneficial gut microbiota. Five species including *Roseburia intestinalis* produce short-chain fatty acids (SCFAs), particularly butyric acid. These SCFAs have been found to protect against intestinal inflammation. The efficacy of anti-programmed cell death protein-1 (PD-1) in CRC can also be improved by inducing functional CD8+ T cells [29–31]. *Clostridium* is one of the most abundant bacterial genera in the human gut. It is primarily responsible for the synthesis of SCFAs, particularly butyric acid [32]. Butyrate is one of the primary fermentation byproducts of butyric acid. It can protect colon cells from the reactive oxygen species (ROS)-induced oxidative stress and stimulate the development of regulatory T cells (Tregs) to improve gut immunological tolerance [33,34]. This study revealed that the abundance of *Clostridium sp-CAG302*, *Clostridium sp-CAG253*, and *Clostridium sp-CAG122* in the digestive tracts of LST patients decreased dramatically. In addition, *Prevotella sp-S7-1-8*, *Firmicutes bacterium-CAG321*, and *Butyricicoccus sp-OF13 − 6* belong to potential probiotic phyla and SCFA-producing genera (Firmicutes, *Prevotella*, and *Butyricicoccus* [35–37]. The significant decrease in their abundance indicated that the intestinal SCFA content in LST patients might be reduced, thereby damaging intestinal health. Spearman correlation analysis showed that the decrease in the relative abundances of *Roseburia*, *Prevotella sp-S7-1-8*, *Firmicutes bacterium-CAG321*, and *Clostridium sp-CAG302* was significantly negatively associated with the ABC transporter metabolic pathway (ko02010), biofilm formation-*Escherichia coli* (ko02026), central carbon metabolism in cancer (ko05230), and phosphotransferase system (ko02060) metabolic pathway.

LST also significantly increased the abundance of some bacteria associated with inflammatory bowel disease (IBD), such as *Escherichia*, a genus of Gram-negative bacteria that cause damage to the intestinal epithelial barrier [38,39]. The genera *Ruminococcus*, *Parabacteroides*, and *Bacteroides* contain species *Ruminococcus sp-AM41 − 2AC*, *Parabacteroides sp-AF39 − 10AC*, and *Bacteroides sp-AF25 − 38AC*, respectively; all these bacteria are linked to IBD and have a positive correlation with irritable bowel syndrome [40–42]. Extracellular mouse pathogen *Citrobacter amalonaticus* can colonize the mucosa of the colon, causing tissue injury and inflammation that leads to diarrhoea and colonic crypt hyperplasia (CCH) [43,44]. Although *Limosilactobacillus gorillae* is a member of the probiotic genus *Limosilactobacillus*, its particular effects have not been fully explored yet. It is necessary to confirm the correlation between this bacterium and IBD. In the current study, the abundance of *Escherichia* and *Citrobacter amalonaticus* significantly promoted ABC transporters, *Escherichia* biofilm formation, central carbon metabolism, and phosphotransferase system metabolism in cancer, especially central carbon metabolism in cancer.

Both the gut and serum differential metabolites in the LST patients were associated with cholesterol/choline metabolism (enriched in the pathways related to cholesterol and choline metabolism in cancer, respectively). A complicated relationship between carcinogenic signalling pathways and cancer metabolism was observed in the altered metabolism of cholesterol and choline, which might be a metabolic marker of cancer [45,46]. Studies have shown that this metabolic disorder is connected to the development and occurrence of several malignancies, including CRC and hepatocellular carcinoma. It is distinguished by the atypical variations in the concentrations of glycochenodeoxycholate and choline phosphate [47,48]. This study revealed differences in glycochenodeoxycholate and phosphatecholine, as well as notable alterations in the gut and serum metabolism in the LST patients. Both phosphocholine and glycerophosphatidylcholine were found to significantly inhibit the growth and invasive ability of colorectal cancer cells by in vitro cellular findings. Arginine biosynthesis, central carbon metabolism in cancer, and butanoate metabolism pathways were considerably enriched. Tumours and malignancies disrupt the amino acid and butyrate metabolism, which is an important phenomenon in intestinal disorders. The abnormalities in the alanine, aspartate, and glutamate metabolism and arginine biosynthesis have been identified as metabolic pathways that might serve as diagnostic indicators for CRC [49,50]. Fumarate, succinate, glutamic acid, glycine, and L-aspartic acid levels increased considerably in both metabolic pathways. Fumarate and succinate are essential metabolic intermediates in the tricarboxylic acid cycle. Fumarate is frequently accumulated as a result of metabolic reprogramming, which promotes the incidence and development of malignancies; therefore, it is classified as a carcinogenic metabolite [51,52]. It was discovered that CRC patients exhibited considerably higher levels of glutamic acid, glycine, and L-aspartic acid. These are significant carbon/nitrogen sources that contribute to the synthesis of critical cell components and might be linked to the ongoing proliferation of cancer cells and energy supply [53,54]. Fumarate, Succinate, and Glutamic acid exacerbate intracellular levels of oxidative stress, promote the expression of key enzymatically active genes in the Choline metabolism in cancer, Cholesterol metabolism pathway (*Chk-alpha*, and *HMG CoA*), and thus promote cancer cell growth. In vitro cellular validation experiments. The changes in these amino acids affect central carbon metabolism in LST patients. In the current study, metagenomic analysis revealed alterations in central carbon metabolism in cancer. Butyrate is widely thought to be healthy; however, the gut microbiota of CRC patients release high levels of butyrate and promote the butyrate metabolic pathway, which plays an important role in maintaining the tumour microenvironment and encouraging tumour growth [53,55].

In conclusion, LST patients have typical disorders of cholesterol metabolism, choline metabolism in cancer, and amino acid metabolism. Glycochenodeoxycholate, fumarate, succinate, glutamic acid, glycine, and L-aspartic acid abnormalities are the biomarkers of LST. Their considerable increase is coupled with a significant decrease in the abundance of probiotics (*Roseburia*, *Roseburia-OF03–24*, *Roseburia intestinalis-CAG13*), which was confirmed using metagenomics and metabolomics. Fumarate, Succinate, and Glutamic acid showed the ability to promote the growth and invasion of cancer cells.

However, this study is restricted to a single-centre design. Future research should include multi-geographical and multi-centre clinical trials to gather a more diverse patient population, enabling a comprehensive analysis of the differences in flora and metabolites across various subtypes. Collaborative efforts are essential for establishing a robust big data platform, which will elucidate the patterns of change in intestinal flora and related metabolites in LST patients. Additionally, our team plans to enrol more LST patients and enhance follow-up investigations and clinical sample collection. This will facilitate a better understanding of whether the altered intestinal flora and metabolites normalize post-tumour resection, thereby validating their role in LST development.

## Conclusions

This study revealed substantial alterations in the gut microbiota of LST patients, including a reduction in the number of SCFA-producing and anti-inflammatory intestinal probiotics as well as an increase in the abundance of pathogenic bacteria capable of causing IBD. Metabolomic analysis revealed that fumarate, succinate, glutamic acid, glycine, and L-aspartic acid levels were considerably higher in the LST patients, altering amino acid metabolism and induce cancer cell growth. Furthermore, the gut and serum metabolomics revealed substantial variations in choline metabolism in cancer and cholesterol metabolism pathways in the LST patients. The multi-omics analysis suggested a potential mechanism by which the microbiota was linked to the progression of LST and provided potential novel targets for the early detection and management of LST, which might serve as a reference for CRC prevention.

## Supplementary Material

Supplementary material 1.docx

## Data Availability

The data associated with this article is openly available at figshare at “https://doi.org/10.6084/m9.figshare.27073486.” The original data of metagenomic were uploaded at NCBI (https://www.ncbi.nlm.nih.gov/bioproject/PRJNA1212478).
