# Supplementary material for: Comprehensive analysis of faecal metagenomic and serum metabolism revealed the role of gut microbes and related metabolites in detecting colorectal lateral spreading tumours
Source: Virulence. 2025 Apr 13;16(1):2489154. doi: 10.1080/21505594.2025.2489154 (PMC12005448; doi:10.1080/21505594.2025.2489154)
Supplement: Supplementary material 1.docx [file KVIR_A_2489154_SM5812.docx]

**Comprehensive analysis of fecal metagenomic and serum metabolism revealed the role of gut microbes and related metabolites in detecting colocolorectal lateral spreading tumors**

Hao Lin ^1, 2, 4†^, Yudai Chen ^1, 3, 4†^, Ming Zhou ^1, 2, 4†^, Hongli Wang ^1, 2, 4^, Lichun Chen ^1, 2, 4^, Li Zheng ^1, 2, 4^, Zhixin Wang ^1, 2, 4^, Xiaoling Zheng ^1, 3, 4*^, Shiyun Lu ^1, 2, 4*^

1. *Shengli Clinical Medical College, Fujian Medical University, Fuzhou, 350000, Fujian, China.*

2. *Department of Gastroenterology, Fujian Provincial Hospital, Fuzhou, 350000, Fujian, China.*

3. *Department of Digestive Endoscopy, Fujian Provincial Hospital, Fuzhou, 350000, Fujian, China.*

4. *Fuzhou University Affiliated Provincial Hospital, Fuzhou, 350000, Fujian, China.*

†These authors contributed equally to this work.

***Corresponding authors:**

Xiaoling Zheng

Email: xiaolingzheng@fjmu.edu.cn; Tel: +86 591 88218888

Shiyun Lu

Email: lushiyun@fjmu.edu.cn; Tel: +86 591 88218888

**1 Propensity score matching analysis**

**1.1 Propensity score matching of baseline data before and after analysis**

This study included 63 LST patients and 85 healthy volunteers, recruited between May 2023 and August 2023. To mitigate the potential impact of confounding variables on the baseline characteristics, propensity score matching (PSM) was employed using SPSS 26.0. The control and LST groups were designated as 0/1 categorical variables. Age, gender, and BMI were treated as covariates, with a 1:1 matching ratio, a caliper value of 0.02, and the calculation of propensity scores. Ultimately, cases with the closest propensity scores were paired. Thirty-five LST patients were successfully matched 1:1 with controls, while 28 LST patients could not be matched and were excluded from further analysis.

Table 1 presents the differences in baseline characteristics between the LST and healthy control (HC) groups both before and after PSM. Prior to matching, the LST group consisted of 27 males (42.9%) and 36 females (57.1%), while the HC group comprised 32 males (37.6%) and 53 females (62.4%). There were no significant differences between the groups with respect to gender, BMI, hypertension, diabetes, family history of colorectal cancer, smoking history, or alcohol consumption. However, age differences were significant. After PSM, there were no significant differences in baseline characteristics between the two groups (*P*>0.05).

Table 1 Baseline data of the LST group and HC group

|  | Before PSM | | | After PSM | | |
| --- | --- | --- | --- | --- | --- | --- |
|  | LST group  (n = 63) | HC group  (n = 85) | *P-*value | LST group  (n = 35) | HC group  (n = 35) | *P-*value |
| Gender, n (%) |  |  | 0.522 |  |  | 0.434 |
| male | 27（42.9） | 32（37.6） |  | 12（34.3） | 9（25.7） |  |
| female | 36（57.1） | 53（62.4） |  | 23（65.7） | 26（74.3） |  |
| Age | 60.0±13.4 | 48.2±11.1 | ＜0.001 | 54.1±12.5 | 54.5±11.9 | 0.892 |
| BMI (kg/m^2^) | 23.3±3.7 | 23.4±3.1 | 0.890 | 23.3±3.9 | 22.9±2.6 | 0.614 |
| Hypertension, n (%) |  |  | 0.464 |  |  | 0.526 |
| No | 48(76.2) | 69(81.2) |  | 28(80.0) | 30(85.7) |  |
| Yes | 15(23.8) | 16(18.8) |  | 7(20.0) | 5(14.3) |  |
| Diabetes, n (%) |  |  | 0.833 |  |  | 1 |
| No | 57(90.5) | 76(89.4) |  | 33(94.3) | 33(94.3) |  |
| Yes | 6(9.5) | 9(10.6) |  | 2(5.7) | 2(5.7) |  |
| Family history of colorectal cancer, n (%) |  |  | 0.251 |  |  | 0.164 |
| No | 57(90.5) | 81(95.3) |  | 31(88.6) | 34(97.1) |  |
| Yes | 6(9.5) | 4(4.7) |  | 4(11.4) | 1(2.9) |  |
| Smoking, n (%) |  |  | 0.815 |  |  | 0.495 |
| No | 54(85.7) | 74(87.1) |  | 29(82.9) | 31(88.6) |  |
| Yes | 9(14.3) | 11(12.9) |  | 6(17.1) | 4(11.4) |  |
| Drinking, n (%) |  |  | 0.657 |  |  | 0.759 |
| No | 55(87.3) | 72(84.7) |  | 29(82.9) | 28(80.0) |  |
| Yes | 8(12.7) | 13(15.3) |  | 6(17.1) | 7(20.0) |  |

**2 Factors influencing the development of cancer in LST**

**2.1 Baseline characteristics of study subjects in the LST subgroup**

Pathological tissue samples from 63 cases in the LST group, prior to PSM, were analyzed. Based on tumor risk stratification, two subgroups were formed: low-risk (LR) (low-grade intraepithelial neoplasia, non-tipped serrated lesions without heterogeneous hyperplasia, or proliferative polyps) and high-risk (HR) (high-grade intraepithelial neoplasia, non-tipped serrated lesions with heterogeneous hyperplasia, or intramucosal carcinoma). Table 2 presents the baseline characteristics of both LST subgroups. The t-test revealed no significant differences in gender, age, BMI, hypertension, diabetes mellitus, family history of colocolorectal cancer, smoking history, or alcohol consumption between the two LST subgroups.

Table 2 Baseline data of 63 LST subgroup subjects

| Subjects | 总数  （n = 63） | LR  （n = 50） | HR  （n = 13） | *P*值 |
| --- | --- | --- | --- | --- |
| Gender, n (%) |  |  |  | 0.109 |
| Male | 27(42.9) | 24(48.0) | 3(23.1) |  |
| Female | 36(57.1) | 26(52.0) | 10(76.9) |  |
| Age | 60.0±13.4 | 63.6±6.6 | 59.0±14.6 | 0.272 |
| BMI (kg/m^2^) | 23.3±3.7 | 23.0±3.3 | 23.4±3.8 | 0.718 |
| Hypertension, n (%) |  |  |  | 0.432 |
| No | 48 | 37(74.0) | 11(84.6) |  |
| Yes | 15 | 13(26.0) | 2(15.4) |  |
| Diabetes, n (%) |  |  |  | 0.419 |
| No | 57(90.5) | 46(92.0) | 11(84.6) |  |
| Yes | 6(9.5) | 4(8.0) | 2(15.4) |  |
| Family history of colocolorectal cancer, n (%) |  |  |  | 0.062 |
| No | 57(90.5) | 47（94.0） | 10（76.9） |  |
| Yes | 6(9.5) | 3（6.0） | 3（23.1） |  |
| Smoking, n (%) |  |  |  | 0.309 |
| No | 54(85.7) | 44（88.0） | 10（76.9） |  |
| Yes | 9(14.3) | 6（12.0） | 3（23.1） |  |
| Drinking, n (%) |  |  |  | 0.744 |
| No | 55(87.3) | 44（88.0） | 11（84.6） |  |
| Yes | 8(12.7) | 6（12.0） | 2（15.4） |  |

**2.2 One-way logistic regression analysis**

Following the one-way logistic regression analysis, no significant differences were observed between the two study groups regarding gender, age, BMI, hypertension, diabetes, family history of colorectal cancer, smoking history, or alcohol consumption (*P*>0.05). These findings suggest that gender, age, BMI, hypertension, diabetes, history of malignancy, smoking history, and alcohol consumption are not risk factors for carcinogenesis in LST (Table 3).

Table 3 One-way logistic regression analysis of carcinogenesis of LST

| Subjects | OR_95*CI* | *P*值 |
| --- | --- | --- |
| Gender | 3.077(0.755~12.533) | 0.117 |
| Age | 1.03（0.98~1.08） | 0.272 |
| BMI | 0.97（0.82~1.15） | 0.713 |
| Hypertension | 1.932(0.377~9.9) | 0.429 |
| Diabetes | 2.09(0.34~12.91) | 0.427 |
| Family history of colocolorectal cancer | 2.7(0.83~26.77) | 0.081 |
| Smoking history | 2.2(0.47~10.33) | 0.318 |
| Drinking history | 1.33(0.24~7.53) | 0.745 |
